# Simultaneous Measurement of Curvature, Strain and Temperature Using a Twin-Core Photonic Crystal Fiber-Based Sensor

**DOI:** 10.3390/s18072145

**Published:** 2018-07-03

**Authors:** Tongtong Zhao, Shuqin Lou, Xin Wang, Wan Zhang, Yulei Wang

**Affiliations:** 1 School of Electronic and Information Engineering, Beijing Jiaotong University, Beijing 100044, China; 14111024@bjtu.edu.cn (T.Z.); xin.wang@bjtu.edu.cn (X.W.); 16111022@bjtu.edu.cn (W.Z.); 2 National Key Laboratory of Science and Technology on Tunable Laser, Harbin Institute of Technology, Harbin 150080, China; wyl@hit.edu.cn

**Keywords:** optical fiber sensor, photonic crystal fiber, twin-core fiber

## Abstract

A novel twin-core photonic crystal fiber-based sensor for simultaneous measurement of curvature, strain and temperature is proposed. The fiber sensor is constructed by splicing the homemade twin-core photonic crystal fiber between two segments of single mode fiber. Affected by the coupling between two cores, the transmission spectrum of the fiber sensor has different wavelength responses to curvature, strain, and temperature. The maximal sensitivities to curvature, strain and temperature are 10.89 nm/m^−1^, 1.24 pm/με and 73.9 pm/°C, respectively. Simultaneous measurement of curvature, strain and temperature can be achieved by monitoring the wavelength shifts of selected valleys in the transmission spectrum. Contrast experiment based on traditional twin-core fiber is carried out. Experimental results demonstrate that twin-core photonic crystal fiber-based sensor has higher sensitivity and better linearity than traditional twin-core fiber-based sensor.

## 1. Introduction

Optical fiber sensors, which have attracted particular attention in recent decades due to their specific advantages of flexibility, high sensitivity, compact size, low cost, and fast response, have been widely used in many sensing application fields [1,2]. Generally, fiber optic sensor obtains the measurement of strain, temperature, curvature, refractive index, and other quantities through measuring the change of intensity or wavelength shift. So far, lots of optical fiber sensors including fiber Bragg gratings (FBGs) [3,4], long-period fiber gratings (LPGs) [5,6], twin-core fiber (TCF) [7,8,9,10], multimode fiber-based Mach-Zehnder interferometer (MZI) [11], tapered fiber-based MZI [12] and the interferometers based on photonic crystal fibers (PCFs) [13,14,15], have been proposed and demonstrated in actual measurement applications. Among them, the TCF-based sensor is widely applied in optical fiber sensing due to its remarkable advantages, such as low cost, great flexibility, and stability.

Compared with other fiber sensors, TCF-based sensors usually show cross-sensitivity to multi-parameters, such as temperature, pressure, strain, and curvature, which is a drawback for the development of optical fiber sensors [16]. However, several researchers take advantage of the cross-sensitivity to obtain simultaneous measurements of dual parameters. In 2009, Kim et al. [17] demonstrated an in-line MZI based on twin-core photonic crystal fiber (TC-PCF) and conducted strain measurements. The proposed MZI also provided a good performance for use as a curvature sensor. In 2010, an all-fiber MZI using suspended TCF was proposed by Frazao et al. [18]. Two interferometers were obtained when the fiber was illuminated by a polarized light. Due to the birefringence of the fiber cores, different sensitivities were observed by applying curvature and temperature. In 2012, an in-line fiber pressure sensor based on TC-PCF was proposed [19]. The temperature response for this sensor has also been measured in the experiment. Recently, Kang et al. [20] proposed a TCF-based fiber laser sensor for measuring temperature and strain. According to the reports, TCF-based sensors are mainly used for single or dual-parameter measurements. However, simultaneous measurement of multiple environmental parameters is necessary for application in fields of oil and gas exploration, oil pipelines, constructional engineering, and bridge engineering. Therefore, there is an urgent need to develop sensors for monitoring multi-parameter simultaneously.

To obtain multi-parameter measurements, we propose a TC-PCF-based senor for simultaneous measurement of curvature, strain, and temperature. In the homemade TC-PCF, two cores are introduced by replacing two air holes with germanium-doped rods. One core is arranged at the center of TC-PCF to easily align with the core of the SMF. Another core is placed off the axis of the TC-PCF to enhance the bending response for curvature measurements. The curvature sensitivity of the device can be achieved as high as 10.89 nm/m^−1^ ranging from 3 m^−1^ to 6.5 m^−1^. The sensor is also experimentally demonstrated to be sensitive to temperature and strain. The sensitivity of strain is 1.24 pm/με in the range from 0 με to 5000 με and the temperature sensitivity is 73.9 pm/°C in the range from 20 °C to 80 °C. Due to the significant linear measurement characteristics, the simultaneous detection of the curvature, strain and temperature can be obtained.

## 2. Fiber Structure and Sensor Design

**Figure 1:**
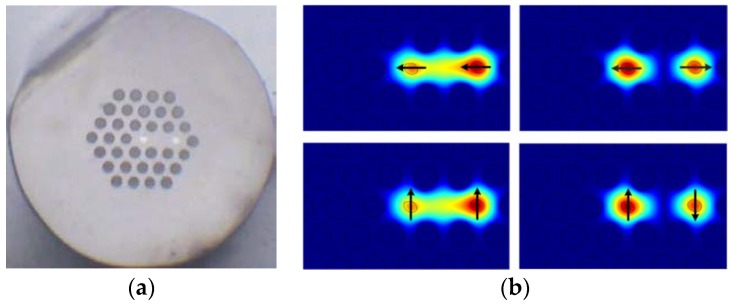
The cross section of homemade TC-PCF (**a**), mode field distribution of the even mode and odd mode in *x*- and *y*-polarization (**b**).

The mode can be confined tightly due to air-hole cladding, which leads to larger coupling coefficients and shorter sensor length. In addition, the introduction of the air holes makes the fiber more sensitive to environmental parameters [19]. Thus, a TC-PCF is designed based on an existed traditional TCF [8]. The diameter of the core in traditional TCF is 3.2 µm and the distance between the dual cores is about 14.2 µm. The refractive index difference of the core and background material is 0.296%. Figure 1a shows the cross-section of the homemade TC-PCF. The fiber structure is constructed by using the similar twin cores with the traditional TCF and the periodic cladding structure of PCF. Two cores are introduced by replacing two air holes with germanium-doped rods. The air hole between two cores is replaced by pure silica rod to enhance the modal coupling. The cladding diameter of the fiber is about 125 μm. The hole pitch and the average hole diameter are 7.5 μm and 5.25 μm, respectively. The diameter of the germanium-doped core is 3 μm. The refractive index difference between the germanium-doped core and background material is about 0.3%.

The modal properties of the homemade TC-PCF can be analyzed by using the full vector finite element method which has been widely used for modeling microstructure fiber. The fiber geometry is obtained by extracting the cross-section image of the homemade TC-PCF sample. According to the coupled mode theory, there are four fundamental supermodes existing in TC-PCF, including *x*-polarized odd mode, *x*-polarized even mode, *y*-polarized odd mode, and *y*-polarized even mode. The mode field distribution at the wavelength of 1550 nm is shown in Figure 1b. Air holes and cores are slightly deformed when the fiber is fabricated, which leads to asymmetrical mode-filed distribution.

**Figure 2:**
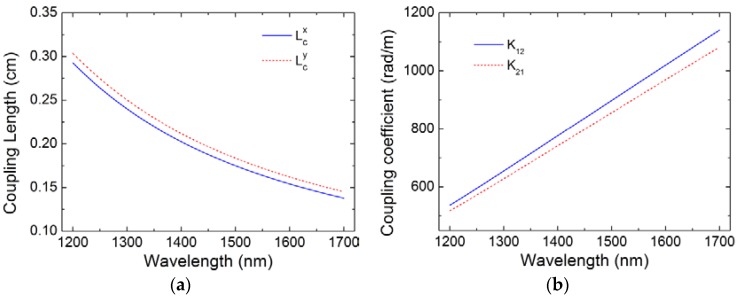
Calculated coupling length (**a**) and coupling coefficient (**b**) for TC-PCF.

By applying the coupled mode theory [21], the coupling length and coupling coefficients of two cores as functions of wavelength are calculated results are shown in Figure 2. The mode fields extend further away from the core when the wavelength increases, which leads to a decrease of the coupling length and an increase of coupling coefficient. The coupling length of *y*-polarized mode is longer than that of *x*-polarized mode because the introduction of two Ge-doped cores and fabrication deviation of the fiber lead to asymmetrical index distribution. The length of fiber coupling changes with wavelength. Therefore, according to the coupled mode theory, the energy in the two cores at the output port varies with wavelength when the fiber length is fixed. Thus, the homemade TC-PCF can be used as a fiber filter. In addition, note that the relationship between the coupling coefficient and wavelength is approximately linear in the wavelength range from 1200 nm to 1700 nm.

**Figure 3:**
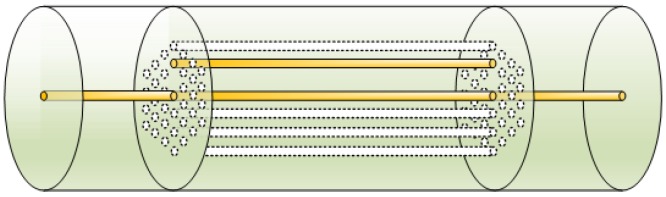
Schematic diagram of the TC-PCF-based sensor.

A sensor can be constructed by splicing the homemade TC-PCF between two segments of SMF. Figure 3 illustrated the schematic diagram of the TC-PCF-based sensor. The core of SMF is aligned to the central core of TC-PCF.

Assuming that input power is launched into one core, the transmitted light intensity of the TC-PCF can be expressed by [21]

(1) $$P_{A}\left(z=L_{0}\right)=1-\frac{K_{12}K_{21}}{\delta^{2}+K_{12}K_{21}}\sin^{2}\left(\sqrt{\delta^{2}+K_{12}K_{21}}L_{0}\right)\;P_{B}\left(z=L_{0}\right)=\frac{K_{12}K_{21}}{\delta^{2}+K_{12}K_{21}}\sin^{2}\left(\sqrt{\delta^{2}+K_{12}K_{21}}L_{0}\right)$$

where *L*_0_ represents the propagation distance. *δ* is related to the refractive index difference between the two cores and can be defined as (*β*_1_ − *β*_2_)/2, where *β*_1_ and *β*_2_ are propagation constants of core modes in two cores.

It can be deduced from Equation (1) that the power transmission in TCF-based sensor is a periodic function. The transmission peaks occur when the following phase condition is satisfied.

(2) $$\sqrt{\delta^{2}+K_{12}K_{21}}L_{0}=\{\begin{array}{cc} n\pi & \mathrm{Core}1\\ \left(n+1/2\right)\pi & \mathrm{Core}2 \end{array}$$

where *n* is integers. The difference of the adjacent peak wavelengths, which can be defined as the free spectra range (FSR), can be calculated from [22]

(3) $$\Delta \lambda =\lambda_{n}-\lambda_{n-1}\approx \frac{\pi }{L_{0}\frac{\partial K\left(\lambda \right)}{\partial \lambda }}$$

which indicates that the FSR is inversely proportional to both fiber length and the derivative of the coupling coefficient *K*. The relationship between coupling coefficient *K* and wavelength is linear, as shown in Figure 2, which means the differential coefficient ∂*K*/∂*λ* is a constant. Thus, the FSR of TC-PCF-based sensor is mainly determined by the fiber length.

**Figure 4:**
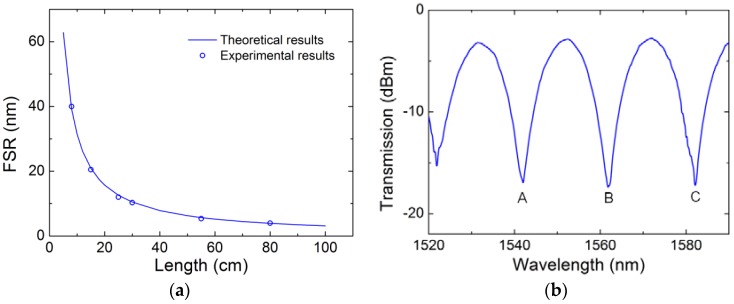
Measured and calculated FSR for different length of TC-PCF (**a**) and the transmission spectra of the 15 cm long TC-PCF sensor (**b**).

In the experiment, several TC-PCF-based sensors with fiber length of 8 cm, 15 cm, 25 cm, 30 cm, 55 cm, and 80 cm are fabricated. The corresponding FSRs measured experimentally are 39.2 nm, 20.1 nm, 12 nm, 10.3 nm, 5.36 nm, and 4 nm, respectively. Meanwhile, the theoretical value of FSR of TC-PCF-based sensors with different fiber length is calculated by using Equation (3). Theoretical results and experimental results are both shown in Figure 4a. Experiment results are consistent with the theoretical ones. As practical application requires small size and high sensitivity, we chose a TC-PCF-based sensor with a fiber length *L*_0_ = 15 cm as the fiber sensor and the corresponding transmission spectrum is shown in Figure 4b. There are three valleys in the transmission spectrum with the FSR of 20.1 nm and minimal extinction ratio of 15 dB, locating at 1542 nm, 1562 nm, and 1582.2 nm, respectively.

## 3. Experimental Setup and Results

**Figure 5:**
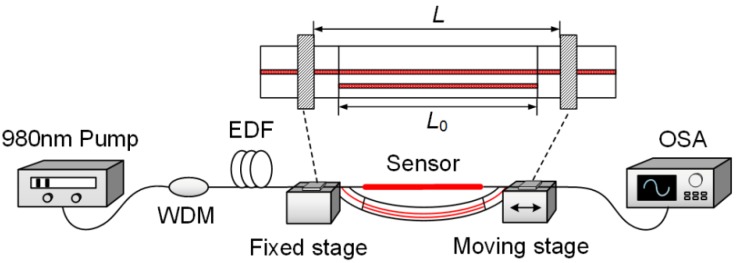
Schematic diagram of the experiment setup.

To measure the transmission characteristics of the TC-PCF, the configuration as shown in Figure 5 is used. A 980 nm pump source with a pump power of 300 mW is injected into the sensor system through a wavelength division multiplexer (WDM). The 980 nm pump source and the erbium-doped fiber (EDF) are employed as the light source. The transmission spectrum of the sensor is detected by an optical spectrum analyzer (OSA, Anritsu, MS9740A) with a resolution of 0.02 nm. To get the largest sensitivity to curvature, the two cores are in the bend plane as shown in Figure 5. The orientation of the TC-PCF is adjusted by looking to the cross section. The 15 cm long TC-PCF-based sensor is fixed by two stages. To protect the splice points between TC-PCF and SMF, the stages are placed away from the splice points. Thus, distance between two stages is set as 20 cm.

**Figure 6:**
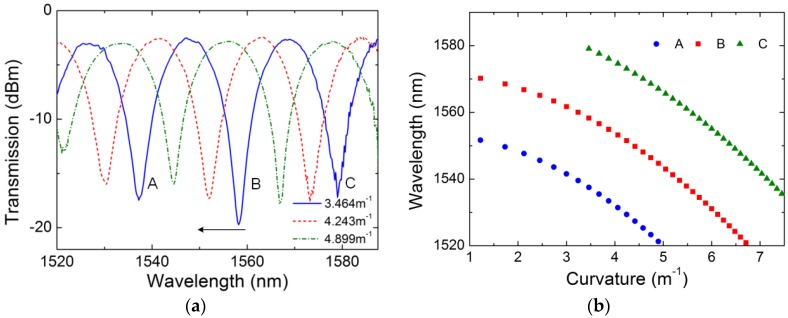

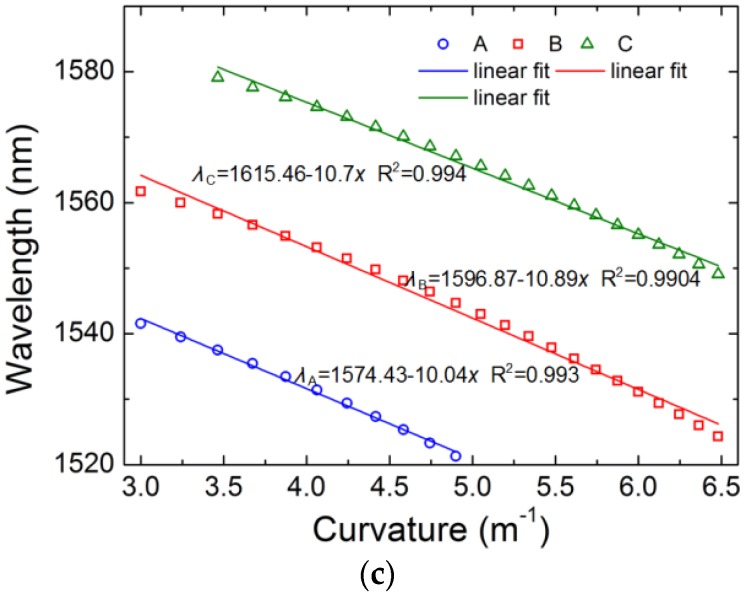
Transmission spectra under different curvature (**a**), wavelength shifts of the valley versus curvature (**b**) and the linear fit of wavelength shifts versus curvature (**c**).

The curvature of the sensor can be changed by manually adjusting the moving stage. The bent fiber is normally approximated as an arc of the circle when the moving stage moves towards the fixed stage. The curvature of the fiber sensor can be calculated as [23]

(4) $$C=\frac{1}{R}≅\sqrt{\frac{24d}{L^{3}}}$$

where *L* and *d* are the initial distance and the movement distance of the moving stage, respectively. Figure 6a shows the transmission spectra of the sensor under different bending curvatures at room temperature (20 °C). The valley shifts towards the shorter wavelength region with the increase of the curvature. Since the refractive index difference between the two cores increases with the curvature of the fiber, the coupling coefficient between two cores should be reduced by reducing the wavelength of the valley according to Equation (2). Figure 6b represents the wavelength shifts at different curvatures, which can be fitted by a second-order polynomial. This is similar with the result of curvatures sensor based on TCF [8]. To achieve simultaneous measurement of multi parameters, significant linear measurement characteristic is needed. Therefore, the measurement range from 3 m^−1^ to 6.5 m^−1^ is chosen to achieve good linearity measurement characteristic for the TC-PCF-based fiber sensor. By using the linear fitting method for the wavelengths of the selected valleys, the relationship between the wavelengths of valleys and the curvature is shown in Figure 6c. In the limited optical spectral ranges, the curvature sensitivity of the sensor is 10.04 nm/m^−1^ for valley A ranging from 3 m^−1^ to 4.9 m^−1^, 10.89 nm/m^−1^ for valley B ranging from 3 m^−1^ to 6.5 m^−1^, 10.7 nm/m^−1^ for valley C ranging from 3.5 m^−1^ to 6.5 m^−1^, respectively.

**Figure 7:**
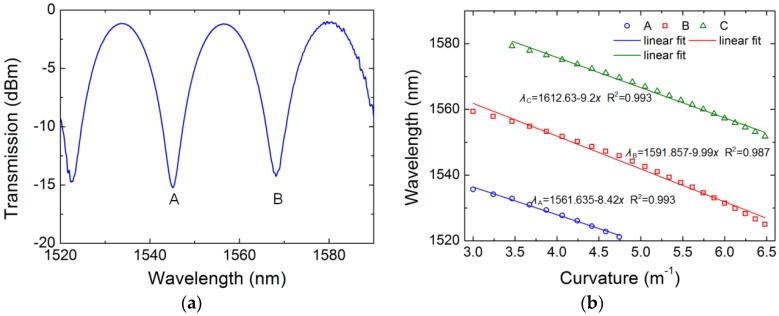
The transmission spectra of the 15 cm long TCF sensor (**a**) and wavelength shifts of the valleys versus curvature (**b**).

A comparison experiment is done by using traditional TCF mentioned above. By splicing the fiber between two segments of SMF, a traditional TCF-based sensor is constructed. The core of SMF is aligned to the central core of TCF, which is same as the TC-PCF-based sensor. The fiber length used in the comparison experiment is 15 cm The transmission spectrum of the sensor is shown in Figure 7a. The FSR of the traditional TCF sensor is larger than that of the TC-PCF sensor due to the difference of fiber structure and structure parameters. Two valleys occur at the wavelength of 1545 nm and 1568 nm in the transmission spectrum of the sensor. The bending characteristics of the traditional TCF sensor are investigated by using the same experimental setup. The relationship between the wavelengths of the valleys and the curvature is shown in Figure 7b. The valley shifts towards short wavelength region, which is same as that of the TC-PCF sensorA third valley appears as the curvature increases. The curvature sensitivities of the three valleys are 8.42 nm/m^−1^, 9.99 nm/m^−1^ and 9.2 nm/m^−1^, respectively. Thus, the TC-PCF-based sensor shows a better performance than the traditional TCF for measuring curvature.

**Figure 8:**
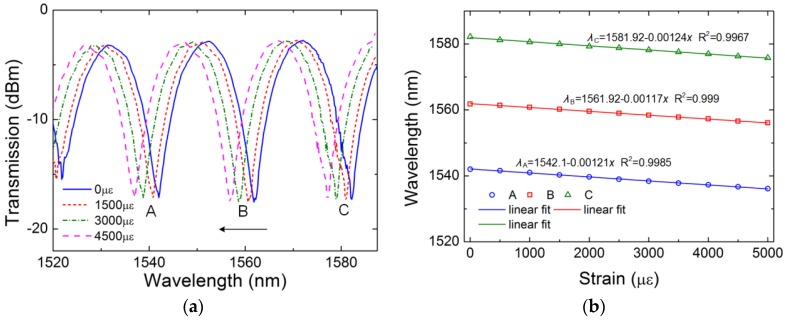
Transmission spectra under different strain (**a**) and wavelength shifts of three valleys versus strain (**b**).

In addition to the high sensitivity of curvature, we also investigate the strain response of the TC-PCF sensor. The fiber length would be increased by Δ*L* and the strain can be represented by *ε* = Δ*L*/*L*. The two stages are adjusted with a step of 0.1 mm, which means that the step of strain is 500 με. The transmission spectra of the sensor under different strains are shown in Figure 8a. The transmission spectrum of the sensor shifts to the short wavelength region with the strain increases from 0 με to 5000 με. According to the elastic-optic effect [24], the refractive index of the core decreases when an axial strain is applied to the TC-PCF. This results in a decrease of the refractive index difference between two cores. Thus, the valley shifts towards the shorter wavelength region with an increase of the strain. Figure 8b shows the linear fitting results. The strain sensitivities of three valleys are 1.21 pm/με at valley A, 1.17 pm/με at valley B and 1.24 pm/με at valley C, respectively.

**Figure 9:**
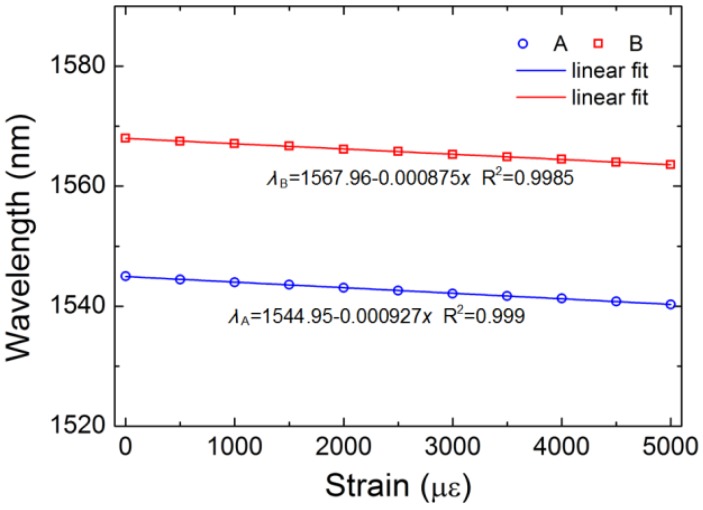
Wavelength shifts of the valleys versus strain.

The strain sensitivity of TCF-based sensor is shown in Figure 9. It can be found that the valleys shift towards shorter wavelength region as the strain increases, which is same with that of the TC-PCFThe strain sensitivities of the traditional TCF-based sensor are 0.927 pm/με at valley A and 0.875 pm/με at valley B, respectively. The refractive index of the core and background material decreases with an increase of strain while the refractive index of air remains constant. The refractive index difference between the core and cladding decreases and the mode easily leaks into the cladding. Thus, the coupling coefficient between the two cores further increases, which makes the TC-PCF-based sensor have higher strain sensitivity than traditional TCF-based sensor.

**Figure 10:**
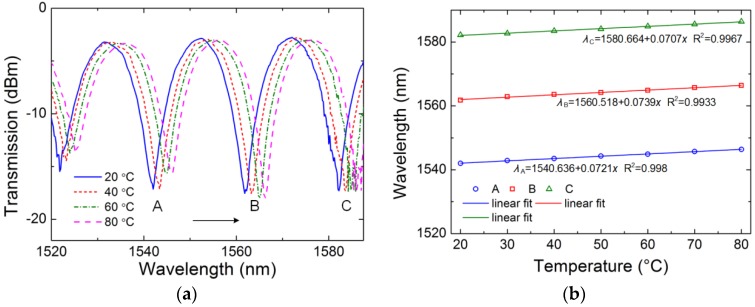
Transmission spectra under different temperature (**a**) and wavelength shifts of the valleys versus temperature (**b**).

At last, the response of the proposed fiber sensor to temperature has been investigated. Figure 10a shows the transmission spectrum shifting with the changing of temperature. The valleys shift to the longer wavelength region along with the increase of temperature, which can be explained by the fact that the germanium-doped and pure silica glass both have positive thermal expansion coefficients. The thermal expansion coefficient of the germanium-doped core is higher than that of pure silica. The refractive index difference between two cores increases with the increase of temperature, which makes the coupling coefficient decrease. Thus, the wavelengths of the valleys shift to a longer wavelength region. The linear relationship between the temperature and the wavelength of valley is shown in Figure 10b. The temperature sensitivities are 72.1 pm/°C, 73.9 pm/°C, and 70.7 pm/°C at the valleys of A, B and C in the range from 20 °C to 80 °C, respectively.

**Figure 11:**
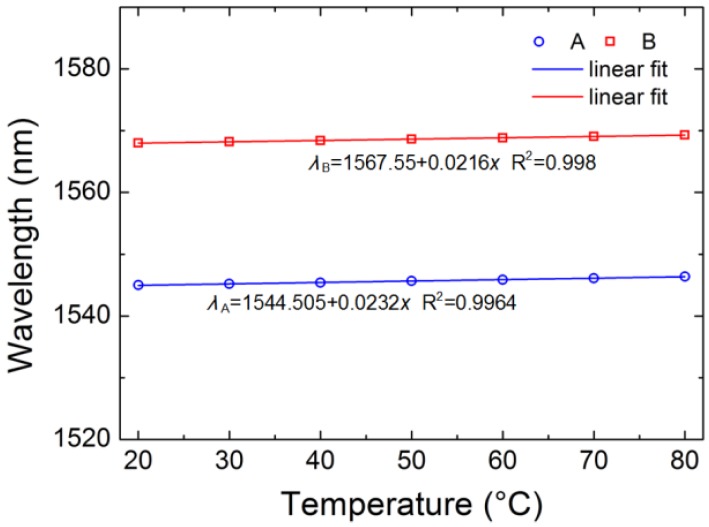
Wavelength shifts of the valleys versus temperature.

A comparison experiment is done by using traditional TCF in the range from 20 °C to 80 °C. It can be seen from Figure 11 that the temperature sensitivities are 23.2 pm/°C and 21.6 pm/°C at valley A and B, respectively. The temperature sensitivity is lower than that of TC-PCF. This is because the Ge-doped rate of the TC-PCF is slightly higher than that of traditional TCF. In addition, the refractive index of the air in the cladding remains constant when the refractive indices of both the Ge-doped core and the silica cladding increase with an increase of temperature. Compared to the traditional TCF, the effective refractive index of the cladding of TC-PCF is insensitive to temperature. The refractive index difference between the cladding and the core increases, which leads to a further decrease of the coupling coefficient between two cores. High refractive index difference between the core and background material and high air-filling fraction of homemade TC-PCF make the thermal sensitivity of TC-PCF higher than that of traditional TCF.

It can be seen from the experiment results that the TC-PCF-based sensor has higher sensitivities and better linearity than a traditional TCF-based sensor. In addition, the transmission spectrum of the sensor will shift when the curvature, strain and temperature are applied to the TC-PCFThe proposed TC-PCF-based sensor has different wavelength responses to these physical parameters at different interference valleys. In addition, the wavelengths of the valleys are linearly shift with these three physical parameters. Therefore, the resulting wavelength shift is a linear superimposition when two or more parameters are applied to the fiber sensor at the same time. When the curvature, strain, and temperature are applied to the TC-PCF, the sum wavelength shifts of the valleys depended on changes in these parameters can be defined as [25]

(5) $$\Delta \lambda_{i}=K_{Ci}\Delta C+K_{Si}\Delta S+K_{Ti}\Delta T,i=A,B,C$$

The above equation can be written in a coefficient matrix

(6) $$\left[\begin{array}{c} \Delta \lambda_{A}\\ \Delta \lambda_{B}\\ \Delta \lambda_{C} \end{array}\right]=\left[\begin{array}{ccc} K_{C,A} & K_{S,A} & K_{T,A}\\ K_{C,B} & K_{S,B} & K_{T,B}\\ K_{C,C} & K_{S,C} & K_{T,C} \end{array}\right]\left[\begin{array}{c} \Delta C\\ \Delta S\\ \Delta T \end{array}\right]$$

where Δ*C*, Δ*S* and Δ*T* are the variation of curvature, strain, and temperature, respectively. Δ*λ* is the wavelength shift of valley A, B and C. *K_C_*, *K_S_* and *K_T_* are the curvature, strain, and temperature sensitivities, respectively. By substituting the sensitivities for different parameters into above equation, the coefficient matrix can be expressed as

(7) $$\left[\begin{array}{c} \Delta \lambda_{A}\\ \Delta \lambda_{B}\\ \Delta \lambda_{C} \end{array}\right]=\left[\begin{array}{ccc} -10.04 & -0.00121 & 0.0721\\ -10.89 & -0.00117 & 0.0739\\ -10.7 & -0.00124 & 0.0707 \end{array}\right]\left[\begin{array}{c} \Delta C\\ \Delta S\\ \Delta T \end{array}\right]$$

By calculating the inverse matrix, the coefficient matrix of the TC-PCF-based sensor for simultaneous measurement of curvature, strain and temperature can be deduced as

(8) $$\left[\begin{array}{c} \Delta C\\ \Delta S\\ \Delta T \end{array}\right]=\left[\begin{array}{ccc} 1.3235 & -0.5764 & -0.74764\\ -3091.4 & 9253.5 & -6520.97\\ 146.147 & 75.016 & -213.39 \end{array}\right]\left[\begin{array}{c} \Delta \lambda_{A}\\ \Delta \lambda_{B}\\ \Delta \lambda_{C} \end{array}\right]$$

where the wavelength shift Δ*λ* is expressed in nanometers (nm), Δ*C*, Δ*S* and Δ*T* are in m^−1^, microstrain (με) and degrees centigrade (°C), respectively. The wavelength shift of each valley can be measured directly. Thus, Δ*C*, Δ*S* and Δ*T* can be calculated by substituting the Δ*λ* into above equation. With this sensitivity matrix, the proposed TC-PCF-based sensor is capable of simultaneously measuring the change of curvature, strain, and temperature by monitoring the wavelength shifts of three valleys in the transmission spectrum.

## 4. Conclusions

A fiber sensor based on TC-PCF, which can be used for simultaneous measurement of curvature, strain, and temperature, is demonstrated. By using coupled mode theory and the equivalent effective index model, the characteristics of TC-PCF has been studied. In the experiment, a 15 cm homemade TC-PCF is spliced into two segments of SMF to form the fiber sensor. The maximal sensitivities to curvature, strain and temperature are 10.89 nm/m^−1^, 1.24 pm/με and 73.9 pm/°C, respectively. Contrast experiment is done by using traditional TCF, and the maximal sensitivities to curvature, strain, and temperature are 9.99 nm/m^−1^, 0.927 pm/με and 23.2 pm/°C, respectively. Experimental results show that the TC-PCF-based sensor has better sensing characteristics than that of traditional TCF. In addition, simultaneous detection of curvature, strain, and temperature can be obtained by monitoring the wavelength shifts of the three selected valleys in the transmission spectrum. The proposed fiber sensor has a great potential to be applied in optical sensing systems due to its high sensitivity, simple structure, and compact size.
